# The Effects of Virtual Reality Telemedicine With Pediatric Patients Diagnosed With Posttraumatic Stress Disorder: Exploratory Research Method Case Report

**DOI:** 10.2196/34346

**Published:** 2023-12-22

**Authors:** Erin Bogdanski

**Affiliations:** 1 TheraVR Inc Emeryville, CA United States

**Keywords:** virtual reality, psychology, neuroscience, behavioral health, telehealth, eHealth, telemedicine, trauma, traumatic, PTSD, posttraumatic stress disorder, mental health, mental condition, mental illness, cognitive behavioral therapy, CBT, avatar, case study, pediatric, child, youth, psychiatric disorder

## Abstract

**Background:**

Trauma-focused cognitive behavioral therapy (TF-CBT) strategies are common interventions to treat child trauma and a posttraumatic stress disorder (PTSD) diagnosis in children with histories of sexual and physical abuse. With the advent of COVID-19, the disruption of child development combined with intense exposure to technology and screen time indicate a need for delivering other novel approaches to treat pediatric PTSD. Virtual reality (VR) has been used with evidence-based TF-CBT as an intervention in lab-based settings, but never as telehealth. Such technologies, including a VR head-mounted device (HMD) programmed with novel TheraVR software, for psychotherapy and treating trauma-related symptoms could redefine how pediatric populations respond to treatment.

**Objective:**

The aim of this exploratory single-case study was to reflect symptom improvement and patient engagement using VR as telehealth.

**Methods:**

The patient was a 10-year-old girl of Middle Eastern descent diagnosed with trauma and comorbid medical conditions. The patient was in divorced joint parental custody and a Child Protective Services report was made with referral for therapy. Night terrors, hallucinations, depression, anxiety, isolation, and encopresis symptoms were assessed at the beginning of treatment. Clinical analysis met the criteria for a diagnosis of early onset PTSD, which was treated over the course of 7 months using TF-CBT. A cross-analysis design was used to compare improved effectiveness in treatment and patient outcomes when moving from delivery of care with telehealth using desktop and tablet synchronous technology to 2D VR desktop telehealth with TheraVR software and subsequently HMD VR telehealth with TheraVR software. Sessions were conducted in private practice providing psychotherapy for remote patient care, collateral care with the family, and coordination of clinical care with the patient’s pediatrician. Safety and protocols for reducing triggers were clinically monitored by the provider.

**Results:**

Over the course of treatment, and moving from standard telehealth to 2D VR to TheraVR with a standalone HMD, there was a significant reduction in PTSD symptoms. The transfer from using the standard video conferencing with face-to-face video to using customizable avatar technology with an assigned scene environment presented an increase in patient retention and follow-through with the treatment goals. The continuous use of delivery of care using VR with the TheraVR software demonstrated breakthrough clinical observations where the patient devised her own interventions for coping with mood, emotional regulation, and negative cognitive processes using the 10 different VR environments.

**Conclusions:**

This study shows the potential efficacy in using VR specifically for younger populations as a better modality of pediatrics care, while improving engagement with the provider through telehealth. These findings suggest the value of further research through larger clinical trials including pediatric patients diagnosed with severe trauma or trauma-related symptoms to assess the effectiveness of TheraVR software.

## Introduction

The delivery of care using telemedicine as synchronous and asynchronous technology for behavioral health has exploded since the advent of the COVID-19 pandemic. The health development of pediatric populations, including young adults, was disrupted due to the shelter-in-place mandate, which created a surge in child and adolescent mental health issues. Additionally, these populations were oversaturated in the usage of screen time and video conferencing for schooling, education, and social engagement [1]. With the goal of understanding the unbalanced usage of screen time with possible negative effects in treating pediatric populations, the purpose of this clinical case study was to challenge the efficacy of using standard telepsychiatry compared to using virtual reality (VR) telehealth for the delivery of mental health care in pediatrics. Specifically considering pediatric populations who developed increased trauma-related symptoms, the goal of this single-case study was to discover if using VR as an evidence-based treatment for trauma-focused cognitive behavioral therapy (TF-CBT) could be a better method of care than standard telehealth (ie, video conferencing) to reduce symptom pathology.

According to novel technology, TheraVR software was designed for use in telehealth, including evidence-based treatments such as TF-CBT with customizable avatar technology in 10 different clinical environments using a head-mounted device (HMD) [2]. The concept of using social VR was to suggest possible better patient outcomes for reducing anxiety, emotional regulation, and physical symptoms related to trauma vagal activity. Comparing stationary video conferencing to using six degrees of freedom (6DOF) allowed the patient to obtain more mind/body clinical benefits in combination with TF-CBT. This paper thus highlights both the clinical and medical extended reality benefits of using VR telehealth with the TheraVR software for treating trauma with pediatric populations compared to standard 2D telehealth methods. The findings show improved outcomes with treatment, patient engagement, and a reduction in symptoms, highlighting the benefits of VR for medical use in terms of enhancing both patient (pediatric) outcomes and parental support [3]. This case report can therefore support future research into pediatric psychology by improving the standards of telepsychiatry care for pediatric behavioral health using VR [4].

## Methods

### Case Formulation

This was an exploratory case study examining the patient experience and clinical outcomes following use of VR telehealth with TheraVR software in treating a 10-year-old girl presenting complex trauma symptoms with comorbid encopresis and night tremors. This case study is based on clinical assessment and observations during 7 months of private practice of individual psychotherapy using both standard telehealth starting with 2D devices for video conferencing and migrating to HMD (Oculus) VR. The TheraVR software with HMD included 6DOF, customizable avatar technology, and evidence-based scene selection. This case study was not designed to provide empirical evidence to support claims of patent or proof of the VR product, but is rather a precursor to the goal of pursuing larger clinical trials for the suggested diagnosis and treatment. Clinical treatment, reports, and observations were conducted and made by the author (EB), a licensed psychotherapist. All names and personal health information have been withheld in this report.

### Patient Case Background and History

The patient was a 10-year-old, who is a first-generation American of Iranian immigrant parents. She was referred to the clinician and author (EB) for private-practice individual psychotherapy services by her pediatrician due to a prior Child Protective Services (CPS) report that had been made by the former provider. Referral for psychotherapy was given by the assigned pediatrician, following a diagnosis of posttraumatic stress disorder (PTSD), social anxiety, and encopresis. The patient’s parents are divorced, with primary custody on the paternal side of the family.

### Diagnostic Assessment

The patient was included in the population of children who were home-schooled due to the COVID-19 pandemic. The patient continued home schooling upon initial intake. Her stepmother contacted the clinician upon initial intake to discuss severe mental illness symptoms reported as nightmares, screaming in her sleep, and encopresis, which were explained within a background of a history of emotional and physical abuse and environmental social withdrawal from peers (due to homeschooling during the pandemic). The patient had split visitation between her biological parents, and primarily lived with the paternal side of the family. There was a clinically reported history of physical and emotional abuse. Although no evidence (eg, photographs) were provided to the clinician, a verified CPS report was made by the pediatrician prior to therapy. The patient’s family of origin was first-generation Middle Eastern immigrants to the United States, who are of the Muslim faith and follow traditional Islamic culture and community practices (eg, Ramadan).

There was no report of an existing history of mental illness or substance use disorder within the family units. Upon consultation with assigned caregivers and discussion of the CPS report, the case was assigned as mandated direct psychotherapy for treating child PTSD and collaboration of care with an assigned pediatrician. The patient had never been prescribed medications; however, reports of comorbid headaches and gastrointestinal issues were provided upon intake.

Pathology and symptomology of the patient met the criteria for severe trauma, depression, loss of peer and social engagement and activities, adjustment disorder, comorbid medical complaints (headaches, gastrointestinal issues, encopresis), and internal family conflict between divorced parents; the possibility of acculturation issues (ie, ethnic and religious aspects, bullying at school) influencing the trauma symptoms was ruled out. Rather, the trauma manifested due to the parental abuse and child development was impaired from social, emotional, and academic aspects due to forced lifestyle and environmental factors during the pandemic (ie, home schooling, lack of peer support). The parents agreed to psychiatric services and all services were provided over telehealth.

### Treatment and Care Delivery

At the time of intake, all psychotherapy sessions were provided through remote patient access through video conferencing for the initial sessions, which were then moved to VR telehealth. The method of treatment included evidence-based TF-CBT, play therapy, and mindfulness-based stress reduction (see Multimedia Appendix 1). Interventions associated with TF-CBT included psychoeducation, parenting component, relaxation skills, affective modulation skills, cognitive coping skills, processing trauma experiences, in vivo mastery of trauma reminders, conjoint child/parent sessions, safety, and developmental trajectory.

For this single-case study, the only interventions used were individual psychotherapy, relaxation skills, cognitive coping, and processing trauma. Parental psychoeducation was also provided. The treatment goals were to reduce the symptom etymology of trauma, both psychologically and with respect to the polyvagal activity, by emphasizing interventions using mind/body clinical techniques. Weekly 1-hour sessions were provided over telehealth. All data were collected by the clinician in the form of progress notes. No clinical questionnaires, assessments, or testing were provided. Patient feedback was collected at the end of each session to determine and assess clinical improvement and to compare the use case experience between standard telehealth (2D device) to VR.

The suggested treatment plan at the time of diagnosis and assessment was CBT to improve positive thought processes, reduce anxious thoughts, and reduce depressive thinking. Adjunctly, the treatment plan included training and awareness around COVID-19; building resilience; and identifying symptoms of trauma when triggered due to parental conflict, abuse, and bullying. Treatment for encopresis was provided using mindfulness, breathing, progressive muscle relaxation (VR only), and sleep hygiene. A treatment plan to address night tremors was included, which entailed drawing/capturing dreams through art and practicing sleep hygiene. The clinical procedure for this case study involved collecting and documenting all therapeutic progress through clinical notes, observing any changes with negative thought process, and any improvement for symptom reduction over the use of standard telehealth followed by the shift to using VR telehealth.

### Ethical Considerations

This case study did not require human subject research ethics approval from the Institutional Review Board, as this exploratory research study was exempt from any formal ethics review. As the patient is a minor, her parents signed the informed consent form, which included the consent to use any data or feedback for continuing research using VR telehealth with the TheraVR system. Additionally, a telemedicine consent form was signed, which disclosed the delivery of care, training, and education of the VR software, along with the safety protocols for both synchronous video conferencing and VR telehealth. Collateral training on the product was provided to the caregivers, which was monitored at all times by the parent both pre- and postsession, using VR telehealth. The disclosure to use any primary collection of data for both clinical progress and product feedback for research purposes was agreed to in signing the telemedicine consent form.

The final consent form for psychotherapy in understanding the Health Insurance Portability and Accountability Act (HIPAA) and Notice of Privacy Practices was signed with the understanding that all data collected for research purposes were to be maintained anonymous and confidential. No compensation was received for participating in this research.

## Results

### Overview of Clinical Outcomes

The results of this case are separated into the three categories of the devices used in the delivery of care: 2D video conferencing (standard telepsychotherapy), 2D VR (avatar technology with no HMD), and HMD VR telepsychiatry (Table 1). The first four sessions were standard telepsychotherapy sessions plus three more sessions that were intermittently provided over video conferencing; four sessions were then provided using 2D VR (tablet version) and six sessions were provided using the Oculus Quest HMD. Therefore, according to the clinical procedure and delivery of care, the majority of sessions provided involved using the HMD with avatar technology based on the patient’s request. Clinical outcomes of this study were collected through the analysis of clinical documentation (ie, progress notes). In total, seven sessions of standard telepsychotherapy were provided, followed by a total of 10 sessions of VR telepsychotherapy (2D and HMD).

No standard measurement was used. The patient’s feedback was collected at the end of each session, which included an emotional report in reduction of depressive symptoms, anxiety, and PTSD-related symptoms. Additionally, the patient provided feedback of her experience using the VR compared to standard telehealth (see the Patient Perspective statement in the Discussion). Outcomes are based on psychological, neurological, and physical aspects, as well as product patient engagement using VR.

**Table 1:** Overview of the clinical outcomes with the three modalities of delivery of care.

Clinical data	Standard telehealth	2D VR^a^	Oculus VR with HMD^b^
Frequency	7 sessions/60 min	4 sessions/50 min+5 min (asynchronous)	6 sessions/50 min+5 min (asynchronous)
Symptom reduction	Poor minimal outcome	Poor minimal outcome	Outstanding improvement; sans encopresis, night terrors, reduction depression
Intervention efficacy	Poor; patient could not focus over video conferencing	Improvement; avatar technology, virtual exercises in CBT^c^ positive affect, improved emotional response	Outstanding improvement; delivery of interventions, customizing avatar, patient-driven modality for cognitive therapy, and reduction of trauma and anxiety symptoms using VR assets
Provider/patient engagement	Poor	Higher retention with patient	Significant improvement in therapeutic alliance and engagement using the HMD, avatar, and behavioral activation
Patient response	No positive feedback on telehealth use	Positive feedback; enjoyed VR, avatar, and scenery	Positive feedback; fully engaged, focused, and response to treatment

^a^VR: virtual reality.

^b^HMD: head-mounted display.

^c^CBT: cognitive behavioral therapy.

### Standard Telehealth

The first four standard telehealth sessions and the following three intermittent sessions (due to technical difficulties) in standard telehealth provided a limited range of reading the Mental Status Examination, communication, and delivering any breathing/body work (mindfulness-based stress reduction) for the patient. TF-CBT interventions used in the initial stages of treatment involved processing the trauma experience and cognitive coping.

Psychoeducation on trauma and abuse was provided to the paternal side of the family. CBT was successful, as the patient was able to practice and respond to thought stopping, reframing, and identifying negative thought processes. The patient response to discussing her abuse and trauma was fairly limited, although there is sufficient data to support evidence of severe trauma. The symptom pathology includes night terrors, gastrointestinal issues, encopresis, social withdrawal, confusion, and poor concentration. For the clinician, it was more problematic to assess symptoms other than verbal cues, and it was likewise challenging to provide TF-CBT exercises over video conferencing. Patient retention was compromised; the patient also had difficulty keeping her face/head within the frame of the screen and exhibited restless usage of the tablet. Standard telehealth sessions did not provide symptom reduction of gastrointestinal issues or headaches. The patient continued to report dysregulated eating. Encopresis was still indicated in parental reports.

### Telehealth With 2D VR

Four sessions of individual psychotherapy were provided using 2D VR telehealth. The results from these sessions did not show any clinical improvement, but did suggest stronger patient engagement in using an alternative method and delivery of telehealth with VR. The patient was very quick to adopt the product and was intuitively able to choose her avatar, engage with the clinician, as well as proceed with selecting the clinician’s avatar. The patient was also given the opportunity to choose her scene selection for this case study only.

The scenes used for the 2D experience were Alpine, Forest, Shrine, and Tundra. TF-CBT interventions used were affective modulation and cognitive coping. Over the course of using the 2D telehealth, the patient was consistent in choosing a “teddy bear” avatar for psychotherapy. Patient retention was higher using this form of telehealth. The patient did exhibit symptom reduction of anxiety and reported at times that she felt “excited,” thus demonstrating a beneficial use of VR for affective modulation. The patient was able to practice cognitive coping in this platform by identifying and expressing negative thought processes and associating these with feelings of shame from being abused.

The conclusion from these interventions using this patient experience over 2D suggests mood improvement based on alternative methods using avatar technology and scene selection, patient empowerment skills pertaining to the patient-driven selection of the avatar, and neurological improvement of focus and concentration with exercise over scene selection. Using this method, the patient did not show any signs of symptom reduction for encopresis, gastrointestinal issues, or headaches. The patient continued to have frequent visits to the pediatrician.

### Telehealth With HMD-Based VR

#### Overview of Clinical Outcomes Using HMD VR

Six full sessions were provided using VR telehealth with the Oculus HMD. The results of these sessions showed significant cognitive, neurological, physical and bio-psycho changes in the patient, including symptom reduction of trauma and elimination of gastrointestinal symptoms and encopresis. All sessions using VR were delivered as telehealth. The patient was trained and prepped for safety protocols, along with HIPAA compliance and tracking for any physical issues (eg, vertigo, nausea). The TF-CBT interventions used were relaxation skills, affective modulation, cognitive coping, processing the traumatic experience, and in vivo mastery within the TheraVR software. The patient did not report any safety issues using VR. However, the patient did report that the Oculus Quest device felt heavy, and that she had to keep adjusting the head strap to be more comfortable. The patient combined usage of the HMD as standing, moving, and sitting while in session with the researcher.

The patient was able to adapt and intuitively use the VR in the session by successfully meeting the clinician in the waiting room, sharing her desired scene selection, and engaging in psychotherapy treatment during each 60-minute session. Outcomes for clinical symptom reduction and improvement included increased positive mood, physiological sense of calm, emotional response reported as feeling “happy,” and reduction of fear when engaged in behavioral activation. Integration of the VR and psychological clinical observations on the differentiation of using HMD compared to video conferencing were significantly seen in the patient’s responses both psychologically and cognitively.

#### Sessions One and Two

The first two sessions involved training and adjusting to the VR space. The patient adapted to the use of exploring, teleportation, and making eye contact and engagement with the clinician in these sessions. Verbally expressing herself through talk therapy was not impaired and audio/visual signals were continuous. Unexpected clinical observations included in the patient experience were noted. Initial session interventions of TF-CBT included cognitive coping and relaxation skills.

In session one, using the Alpine scene, the patient was engaged in memory association organically, which produced pleasant memories of her past. In combination with the behavioral activation, the patient would climb waterfalls, yet continue to engage in talk therapy. The patient was consistent with selecting a “teddy bear” avatar for most of the sessions. Upon interview and feedback of the avatar selection, the patient reported that this avatar felt “cute and adorable.” Specific TF-CBT interventions used included cognitive coping and processing the traumatic experience. Clinical analysis was observed as loss of emotional attention due to divorced volatile parents. During the course of treatment, the patient was greatly isolated at most times and was confined to her bedroom (due to the pandemic). During therapy, the patient had demonstrated and expressed her withdrawn and avoidant attachment with her mother due to a history of abuse. The patient’s relationship with her biological father shows healthy attachment; however, there is little emotion or affection from the parent. In response to the integration of avatar technology with psychotherapy, this clinical observation through the patient processing her trauma was not evident using standard telepsychiatry. The patient appeared to demonstrate higher levels of trust and rapport while using the avatar technology.

In session 2, the HMD VR with TheraVR software was delivered in the Shrine scene. As per the treatment plan using TF-CBT, the primary intervention used in this session involved relaxation exercises. The clinical outcomes observed and recorded were that the patient reported she felt calm and relaxed. Relaxation exercises and mindfulness meditation were taught by the clinician. Assets in VR used were the waterfall, combined with avatar technology for full-body immersion and behavioral activation. This session produced a remarkable observation of fear reduction. The patient had no feelings of fear jumping from the top of the waterfall to the base without the guidance of the provider. This in vivo experience was later used in the VR sessions to build mastery of discussing her trauma. In the comorbid analysis, there were no reports of improvement or change with gastrointestinal or encopresis issues.

#### Session Three

The HMD-based VR was delivered in the Forest scene for session three. Clinical observations collected in this session were that the patient reported feeling calmer and “happier.” The patient also reported the comparison of being in the forest with the HMD compared to using the 2D experience. Through clinical progress notes, the patient reported that she felt happier in the forest scene than being on the tablet. During the session, TF-CBT interventions were used for cognitive coping and affective modulation. The cognitive and neurological observations of the patient included thought association and memory association triggered by assets within the scene, and the falling leaves exercise (see Multimedia Appendix 2) produced feelings of calm and induced a musical cognitive response (the patient started singing “London Bridge is Falling Down”). The final clinical observation and breakthrough experience observed in this session was behavioral. The patient, in avatar form of a “teddy bear,” embraced the clinician. Clinical analysis is not of disinhibited development, but observed to be the desire to express compassion, loss of social peer support due to COVID-19, and strong attachment issues. This indicates differentiation factors using VR as opposed to standardized telehealth, as no physical engagement can be provided through video conferencing or in-person office visits. No changes or improvements of gastrointestinal issues or headaches were reported in this session.

#### Session Four

The patient changed her avatar selection to a humanoid (female child) and the patient selected the clinician avatar to be a male child. The scene selection in this session was the Desert scene (see Multimedia Appendix 3). Clinical observations were based on the patient’s feedback indicating that the colors and environment felt soothing and the patient expressed that she appreciated the asset of the sunset. Clinical interventions for TF-CBT used were processing the traumatic experience, in vivo mastery of trauma reminders, and developing safety skills. The clinician had the patient project negative thoughts and to virtually put them into one of the assets; she selected a cauldron over a hot fire. The clinical usage involves cognitive imagery to “melt” the abuse and trauma, thought replacement, practice compassion, and thoughts of gratitude with guided imagery to make the “boiling soup” healthy. Patient feedback was that she enjoyed using VR to practice the assigned CBT exercise. The patient’s feedback was that she liked being able to see her negative thoughts disappear in the cauldron. Thus, the clinical analysis was that the VR was an excellent use of the art/play therapy component for TF-CBT. In standard TF-CBT, the patient will express and share the traumatic event through drawing/art. In VR, this was improvised through the use of assets and patient-driven engagement with the VR imagery and objects within the scene. During this time in treatment, the patient’s parents reported she had stopped experiencing encopresis.

#### Session Five

Results from this session further demonstrated the patient’s positive engagement and participation with direct psychotherapy and the significant use of VR assets to induce memory recognition and trigger discussion of her trauma and abuse. Scene selection in this session was the Island, where the patient observed a boat as a trigger to remember past events and recollection of memories. The avatar selection changed with a gender interchange of the provider, in which the patient had chosen the provider to be an elderly Caucasian man. TF-CBT clinical interventions used were processing the trauma experience and cognitive coping. Clinical analysis of this session was concentrated with talk therapy and CBT. A remarkable observation for the telehealth experience in this session was that the patient was starting to make direct eye contact, “mirror neuron,” and exhibited engagement with the clinician. This process developed organically, as seen in direct video conferencing in standard telepsychiatry. Additionally, full retention of patient engagement for standard 60-minute sessions was observed without disruption.

#### Session Six

This was the final session using HMD VR telehealth before the termination of services. The scene selection in this session was the Shrine (see Multimedia Appendix 4) and avatar selection was humanoid, with female child avatars for both the patient and provider. Treatment and clinical interventions for TF-CBT were relaxation exercises and cognitive coping. An outstanding clinical observation during treatment was the patient-driven suggestion of using the clouds to practice relaxation. The patient suggested doing this without the guidance of the clinician based on her immediate response to the immersive scene and her mental state at the time, as she was experiencing anxiety due to school exams. The patient and clinician (in avatar form) kneeled side by side on the grass, and the patient guided the clinician to observe her imagery in the cloud shapes as specific objects (animals, mermaids). The patient would then ask the clinician to find them by pointing her avatar’s finger to the sky. Remarkable clinical and product observation noted is that this engagement was entirely carried out remotely as telehealth, and the avatar technology allowed a dyadic experience of engaging in an activity together as would be performed in an office in person. Critical analysis of this observation is the development of patient empowerment and efficacy while using VR telepsychiatry. The patient was starting to develop her own strengths and desired exercises to help with symptom reduction without any forced intervention. Clinical outcomes of the “cloud busting” exercise included producing feelings of relaxation, anxiety reduction, and calmness.

Upon clinical observation and analysis of this case, it is determined that there was a significant difference in the patient experience going from standard telepsychiatry to using HMD VR telehealth. This observation includes that the change to using VR telehealth provided a significant reduction in gastrointestinal issues, elimination of encopresis, improved cognitive/behavioral skills, identifying and expressing signs of abuse, and improved social coping skills while returning back to school after COVID-19.

## Discussion

### Principal Findings

After careful analysis and comparison of standard telehealth with VR telehealth in this case study, the patient reported and the clinician observed significant symptom improvement/reduction and direct improvement in patient engagement using HMD with TheraVR software as telehealth. It was observed that using standard telepsychotherapy video conferencing does provide an accurate reading of the Mental Status Exam. Although video conferencing enables observing facial cues, there is little or no observation of the body or central nervous system of the patient for biometric tracking. After the initial sessions of using video conferencing, the patient engagement was low and no critical symptoms improved. It may be that the patient’s age, the bio-psycho-social impact of COVID-19, and the overuse of video conferencing in day-to-day life compromised the experience of telepsychotherapy using both a tablet and desktop computer.

### Implications for Practice and Future Research

With respect to the clinician’s experience, it was harder to provide treatment, as the patient’s retention was poor through standard telehealth. Collecting critical information verbally from the patient regarding the history of abuse and trauma was problematic in telecommunications using standard video teleconferencing.

The migration and adaptation to using a VR 2D experience did not produce significant symptom improvement/reduction of encopresis, gastrointestinal issues, or headaches. However, the patient did show higher rates of engagement and exhibited an improved mood using 2D VR. Other observations included positive clinical outcomes following use of 2D VR, along with the patient’s fast adoption, more verbal engagement, willingness to participate in therapy as an avatar, and more verbal disclosure during the therapy sessions. Although improvements in establishing therapeutic alliance and comfort with therapy were observed, clinical reduction of symptoms may not have manifested due to lack of a mind/body experience in vivo with the clinician. The product and clinical response suggest that although this platform may support patient engagement, there may be better platforms for telepsychotherapy using extended reality technologies (ie, VR, artificial intelligence, augmented reality).

The transition from the 2D experience to HMD VR telepsychotherapy produced significant results in observed clinical outcomes, physical symptoms, and patient engagement using this platform. Most critical among these in the long-term use of the product was the follow-through with patient empowerment, efficacy, and change and elimination of comorbid symptoms of encopresis and gastrointestinal issues. Using VR with 6DOF demonstrated physiological effects with the patient during psychiatric treatment as she did report symptom reduction with her stomach and digestive issues after relaxation exercises. Delivery of telepsychotherapy using VR was reported to be clinically beneficial using the combination of avatar technology with scene selection, which showed better retention. With improved delivery of the assigned intervention, TF-CBT, there was development of organically processed psychological experiences in VR that benefited the patient (ie, mirror neurons, self-directed relaxation exercises, practicing compassion and healthy social attachment).

This analysis and observation support the need for further investigation and to pursue large-scale clinical trials testing this technology with children and adolescent populations. Likewise, the limitations of VR telepsychotherapy for remote patient usage must be improved with biometric readings to provide more accurate measurements of the clinical outcomes for psychiatric treatment for trauma-focused interventions (eg, PTSD, long COVID). However, it is suggested that combining psychiatric testing and questionnaires during phase one of VR development will supplement for use cases in the course of the development implementation of heart rate variability (HRV) biometric tracking for remote patient usage.

### Limitations

Improving the delivery of care to a higher medical extended reality headset such as the PICO Technologies or HTC-VIVE headset is expected to improve the clinical capture and ergonomic usage for pediatrics. The Oculus Quest HMD is too heavy for children under the age of 14 years. Software improvements can be implemented by improving the credentials and log-ins to be more user-friendly for children. These aspects were cumbersome and complicated for the child, requiring assistance from the parent, which further complicates the efficiency and urgency of care. However, log-ins/credentials appropriate for adolescents/young adults could resolve this issue. Clinical risks and limitations suggest integrating live streaming and HRV tracking for remote patient care for clinician usage to track the central nervous system response of patients with a severe diagnosis (ie, suicidal ideation and self-harming). This will provide better assessment and reading of the patient during treatment and posttreatment and improve assessments for better treatment planning.

Avatar technology can be improved by integrating more real capture of the providers with customization and by adding additional avatar features to improve interventions (eg, animals, moving leaves, flowers). Suggestions for evidence-based treatment are to focus on specific interventions to provide direct patient treatment and follow-through. Furthermore, more patient-directed exercises using behavioral activation could have better clinical outcomes for treating trauma due to compromised complex polyvagal issues. Integrating mind/body usage with 6DOF could significantly improve evidence-based treatments such as TF-CBT and possibly other interventions for child PTSD.

### Conclusions

To further investigate this use case treatment for child PTSD requires pursuing robust clinical trials for pediatric and youth with severe mental illness without psychosis or Axis I criteria. A cross-analysis of standard telehealth with VR is suggested, which includes avatar customization for patient engagement, the bio-psycho-social effects of 6DOF, and biometrics tracking using HRV to improve treatment outcomes.

Further investigation into both the product development and clinical interventions used and standardized for VR telehealth is suggested in trials and to explore specific populations who will benefit from this form of care delivery. This platform is suggested for the pediatric population in treating trauma, depression, anxiety, and social isolation, and as a preventative tool to address child and youth violence ideation. In conclusion, VR telehealth using avatar technology appears to have a positive psychological benefit in practice for psychotherapy with pediatric populations based on the data and clinical results from this single-case study.

### Patient Perspective

The patient involved in this study was a minor and provided feedback after every therapy session verbally. The significant feedback reported after switching from standard telehealth with the TheraVR HMD experience was positive and clinically effective for the patient. The patient reported that she was happier and calmer, and at times she reported she felt “stronger” being an avatar. Clinically, this suggestion of empowering the patient by allowing them to experience a “second self” could be beneficial for social anxiety reduction with this age group post COVID-19. The patient reported that she felt that she could talk about difficult subjects and felt closer to her clinician. She reported that she felt it was easier to tell her clinician about her trauma.

## Appendix

Multimedia Appendix 1 Mindfulness exercise with virtual reality.

Multimedia Appendix 2 TheraVR mindfulness exercise.

Multimedia Appendix 3 TheraVR cognitive behavioral therapy; Desert scene.

Multimedia Appendix 4 TheraVR mindfulness-based stress reduction; Shrine scene.

## Abbreviations

CPS: Child Protective Services
HIPAA: Health Insurance Portability and Accountability Act
HMD: head-mounted device
HRV: heart rate variability
PTSD: posttraumatic stress disorder
TF-CBT: trauma-focused cognitive behavioral therapy
VR: virtual reality
6DOF: six degrees of freedom
