# Supplementary figures and images for: The Effects of Virtual Reality Telemedicine With Pediatric Patients Diagnosed With Posttraumatic Stress Disorder: Exploratory Research Method Case Report
Source: JMIR Form Res. 2023 Dec 22;7:e34346. doi: 10.2196/34346 (PMC10770779; doi:10.2196/34346)

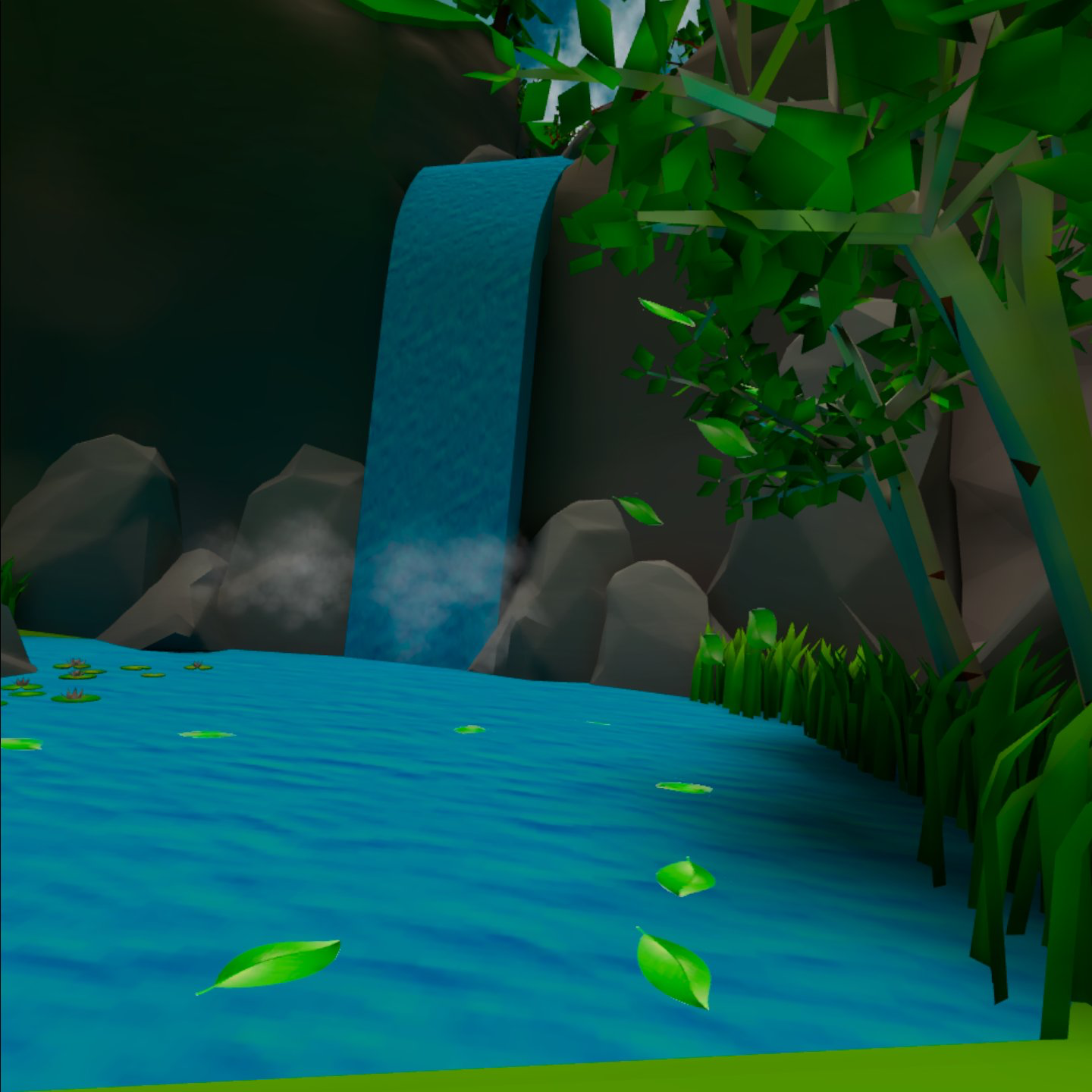

Supplement: Multimedia Appendix 2 [file formative_v7i1e34346_app2.png]

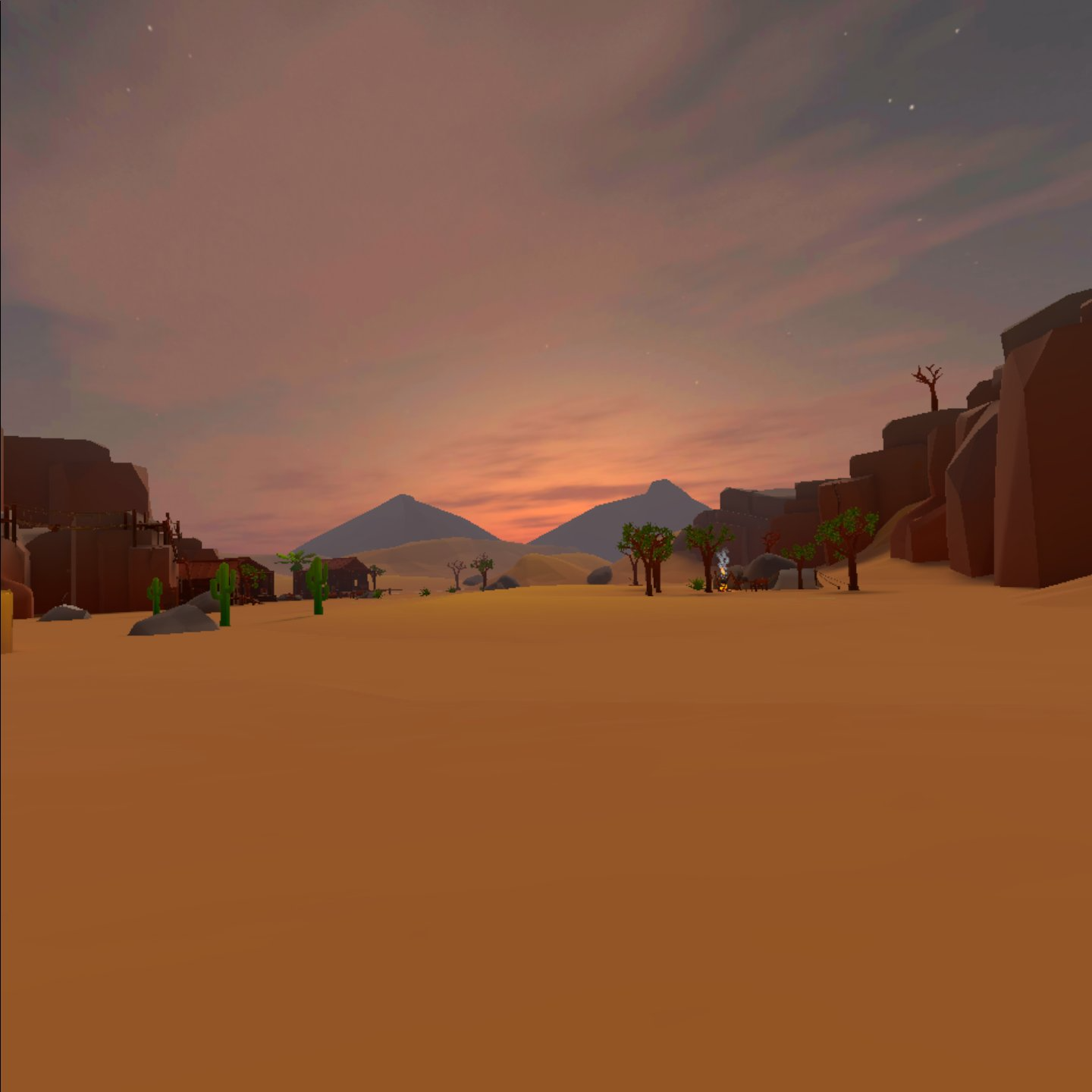

Supplement: Multimedia Appendix 3 [file formative_v7i1e34346_app3.png]

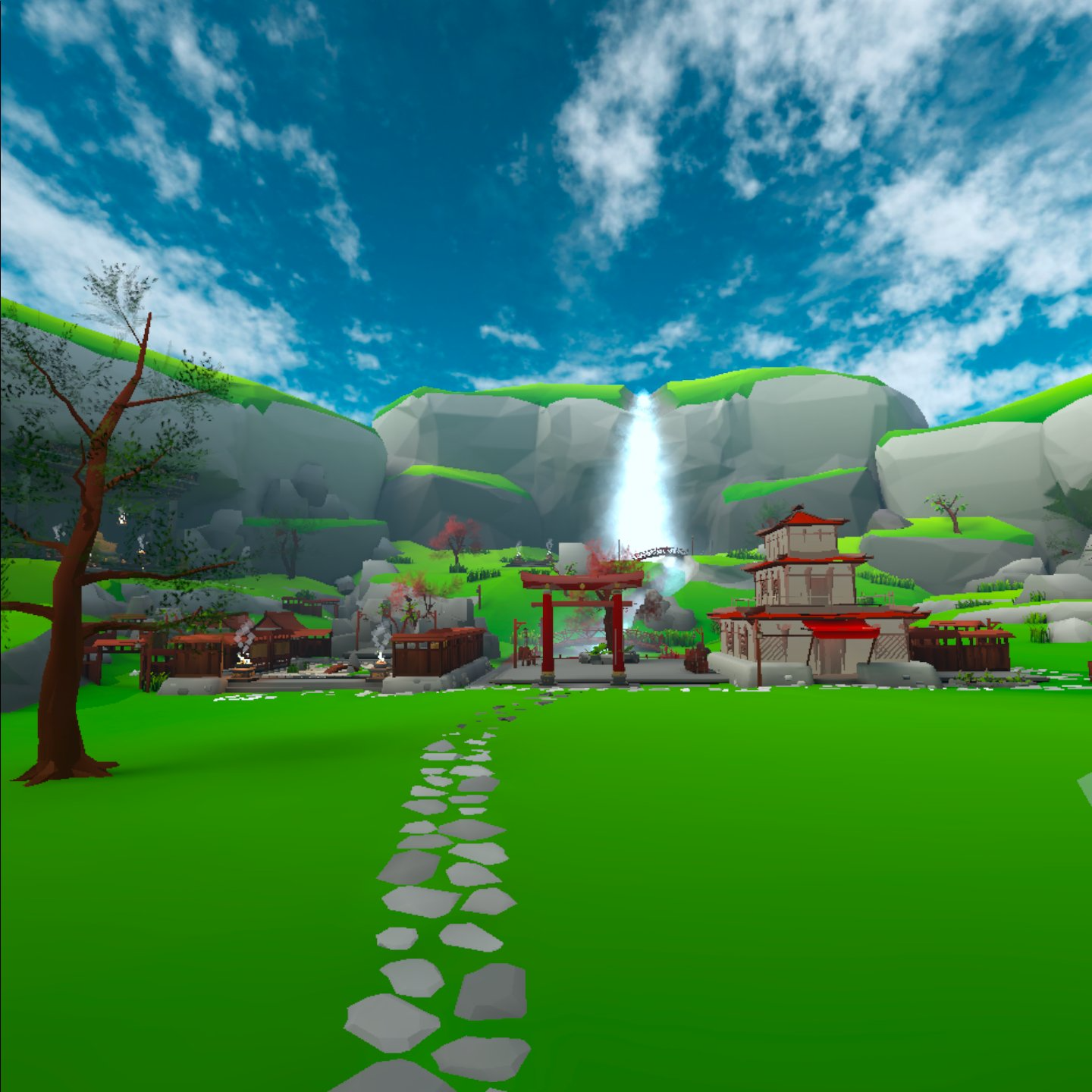

Supplement: Multimedia Appendix 4 [file formative_v7i1e34346_app4.png]
